# MiR-139-5p is a potent tumor suppressor in adult acute myeloid leukemia

**DOI:** 10.1038/bcj.2016.110

**Published:** 2016-12-09

**Authors:** K Krowiorz, J Ruschmann, C Lai, M Ngom, T Maetzig, V Martins, A Scheffold, E Schneider, N Pochert, C Miller, L Palmqvist, A Staffas, M Mulaw, S R Bohl, C Buske, M Heuser, J Kraus, K O'Neill, C L Hansen, O I Petriv, H Kestler, H Döhner, L Bullinger, K Döhner, R K Humphries, A Rouhi, F Kuchenbauer

**Affiliations:** 1 Department of Internal Medicine III, University Hospital of Ulm, Ulm, Germany; 2 Terry Fox Laboratory, British Columbia Cancer Agency, Vancouver, Canada; 3 Department of Clinical Chemistry and Transfusion Medicine, Institute of Biomedicine, Sahlgrenska Academy, University of Gothenburg, Gothenburg, Sweden; 4 Department of Clinical Chemistry, Sahlgrenska University Hospital, Gothenburg, Sweden; 5 Institute of Experimental Cancer Research, Comprehensive Cancer Centre Ulm, Ulm, Germany; 6 Department of Hematology, Homeostasis, Oncology and Stem Cell Transplantation, Hannover Medical School, Hannover, Germany; 7 Medical Systems Biology, Ulm University, Ulm, Germany; 8 Centre for High-Throughput Biology, University of British Columbia, Vancouver, British Columbia, Canada; 9 Department of Physics and Astronomy, University of British Columbia, Vancouver, British Columbia, Canada

Hematopoiesis depends on a tightly controlled balance of self-renewal, proliferation, cell death and differentiation. Although the disturbance of this equilibrium creates a predisposition to leukemogenesis, targeted manipulation or modulation can in turn lead to therapeutic advances. In addition to chromosomal aberrations and frequently mutated genes such as *NPM1* and *FLT3*, dysregulation of microRNAs (miRNAs) is now recognized as having an important role in leukemogenesis. Distinct miRNA expression profiles can classify AML subgroups and miRNAs have recently emerged as novel therapeutic targets in hematopoietic malignancies.^1, 2^

Building on our previous efforts to resolve potential involvement of miRNAs in hematopoiesis, we identified miR-139-5p as a myeloid-specific miRNA with expression being restricted to neutrophils and macrophages^3^ (Supplementary Figure S1A). This distinct expression profile during normal hematopoiesis pointed towards a possible deregulation in malignant hematopoiesis.

In order to relate miR-139-5p expression to distinct genetic subgroups and clinical outcome in adult AML patients, we analyzed the published miRNA sequencing dataset from The Cancer Genome Atlas (TCGA).^4^ A trend for prolonged overall survival (OS) was observed for patients with miR-139-5p levels above the median independent of cytogenetic subgroup (*P*=0.07, Figure 1a left panel), whereas AML patients with normal karyotype (CN-AML) exhibited a significantly better OS (*P*=0.02, Figure 1a right panel). Our findings were further corroborated in CN-AML by comparing the expression quartiles, demonstrating that low miR-139-5p expressors (q3 and q4) are associated with an unfavorable OS (Supplementary Figure S1B). In addition, no effect could be demonstrated for the less abundant miR-139-3p (Supplementary Figure S1C), indicating that miR-139-5p is the active strand. Our findings are in line with Emmrich *et al.*,^5^ who associated high miR-139-5p levels with a favorable outcome in pediatric AML. Further analysis of the TCGA dataset revealed a significant downregulation of miR-139-5p in CN-AML patients harboring mutated FLT3 (*P*=0.03 and *P*=0.001, respectively) compared with the total AML population. (Figure 1b left panel). Considering the poor prognosis of CN-AML patients with mutated *FLT3*,^6^ our findings reinforce the unfavorable outcome of CN-AML patients with low miR-139-5p levels. In contrast, miR-139-5p was upregulated in patients carrying an inv(16) (*P*=0.001) translocation, a prognostically favorable AML subgroup (Figure 1b left panel). No differences were found for miR-139-5p levels in AML patients with t(15;17) and MLL rearrangements, indicating that miR-139-5p levels are associated with specific genetic subtypes rather than with the degree of differentiation. These findings were validated by qRT-PCR in an independent cohort consisting of 49 adult AML patients and 4 healthy donors of total bone marrow (bm) referred to as the Ulm cohort with uniform treatment procedure. All patient samples from the Ulm cohort were collected from adult patients enroled on German-Austrian AML Study Group (AMLSG) treatment protocols for younger adults (AMLSG-HD98A (NCT00146120) and AMLSG 07-04 (NCT00151242)) and comprised Ficoll gradient purified mononuclear cells mainly from diagnostic bm samples with blast counts of ~80% in all analyzed cases. In line with the TCGA dataset, patients harboring mutated *FLT3* displayed the lowest miR-139-5p expression among the various cytogenetic subtypes in the Ulm cohort (*P*=0.03, Figure 1b right panel). Decreased expression of miR-139 in *NPM1*-mutated CN-AML patients, has been previously reported by Garzon *et al.*^7^ We observed a similar trend within the TCGA dataset (*P*=0.06) (Figure 1b left panel), highlighting the robustness of both the datasets. Taken together, we have demonstrated in two independent AML patient cohorts that decreased miR-139-5p levels are associated with mutated *FLT3* and with a lower OS in CN-AML in the TCGA dataset. However, it remains to be determined if miR-139-5p is directly or indirectly transcriptionally regulated through activated FLT3.

The distinct expression patterns of miR-139-5p in CN-AML imply a tumor suppressor role for this miRNA. In order to further explore the therapeutic potential of miR-139-5p, we used a syngenic bm transplantation AML model based on the transformation of murine bm cells through the combined retroviral overexpression of Hoxa9 and Meis1 (Hoxa9/Meis1), which causes a rapid AML *in vivo* compared with the pre-leukemic Hoxa9 cells that lead to a long latency AML.^8^ Upregulation of *HOXA9* and *MEIS1* is frequently observed in *NPM1*-mutated CN-AML and enhances FLT3 signaling.^9, 10^ Based on the expression pattern of miR-139-5p in AML patients, we further hypothesized that miR-139-5p would be downregulated in Hoxa9/Meis1 cells compared with untransformed wild-type bm cells. Indeed, miR-139-5p was significantly downregulated in Hoxa9/Meis1 cells compared with untransduced bm cells as measured by qRT-PCR (Supplementary Figure S1D), highlighting the relevance of this *in vivo* model. To validate the putative tumor suppressor function of miR-139-5p, its expression levels were restored through ectopic lentiviral coexpression of miR-139 and GFP as reported in Hoxa9/Meis1-transformed bm cells. Hoxa9/Meis1/miR-139-5p bm or Hoxa9/Meis1 cells overexpressing an empty control vector (miR-ctrl) were FACS-sorted for GFP, followed by transplantation of 2 × 10^5^ cells per arm into lethally irradiated recipient mice.^10^ Restoration of miR-139 levels significantly delayed Hoxa9/Meis1-mediated leukemogenesis (*P*=0.0003, Figure 2a) in two independent experiments, highlighting its tumor suppressor activity in a primary transplantation model. Bm cells of deceased mice retained overexpression of miR-139-5p compared with the control arm as quantified by qRT-PCR (Supplementary Figure S1E). Lentiviral expression of miR-139-5p in Hoxa9/Meis1 cells restored miR-139-5p at similar levels compared with untransduced bm cells (Supplementary Figures 1D and E), minimizing possible artefacts of ectopic expression. Notably, we detected no endogenous expression of miR-139-3p in the Hoxa9/Meis1/miR-ctrl bm and overexpression of miR-139 generated lower levels of miR-139-3p in comparison with miR-139-5p, highlighting miR-139-5p as the active strand (Supplementary Figure S1F).^11^

Recently, Gibbs *et al.*^12^ demonstrated that Hoxa9/Meis1 cells harbor three, immunophenotypically distinct compartments with varying tumor-initiating activity. Therefore, bm cells of deceased mice were analyzed by flow cytometry for their respective immunophenotype (Figure 2b). We found a significant reduction of c-Kit^+^ and Lin^-^c-Kit^+^ cells in Hoxa9/Meis1/miR-139-5p cells, the subpopulations with enriched leukemia-initiating cells (LICs) frequency (*P*=0.01, Figure 2b) as shown by Gibbs *et al.*^12^ Morphological analysis of cytospins of bm cells from deceased Hoxa9/Meis1/miR-139-5p mice revealed more mature blast cells (Figure 2c left panel) and significantly lower blast counts (*P*=0.01, Figure 2c right panel) compared with the control arm. Hoxa9 and Meis1 cooperate to induce leukemia and are both downregulated during normal myeloid differentiation.^8^ Hoxa9/Meis1-immortalized bm progenitor cells display a resistance against granulocyte-colony-stimulating factor (G-CSF) and granulocyte-macrophage colony-stimulating factor (GM-CSF), resulting in a block of neutrophil, granulocytic and macrophage differentiation.^13^ Our findings suggest that miR-139-5p overexpression might partly overcome this block of differentiation in Hoxa9/Meis1 AML cells *in vivo*. In order to explore the potential of miR-139-5p as an inducer of myeloid differentiation, we retrovirally overexpressed miR-139-5p in 32D cells, an established model of granulocytic differentiation. Increased miR-139 levels promoted G-CSF-induced granulocytic differentiation in 32D cells (Supplementary Figure S1F), supporting our observation in Hoxa9/Meis1/miR-139-5p cells. Mechanistically, it still remains to be determined if miR-139-5p exerts its anti-leukemic effects through restored differentiation and thus modulation of the LIC compartment via repression of EIF4G2, a miR-139-5p target.^5^ In addition, Alemdehy *et al.* highlighted a role for miR-139-3p, the passenger strand of miR-139, in interstrand crosslinks (ICLs), which causes a predisposition for AML.^14^

On the basis of increasing interest in miRNAs as therapeutic targets in AML,^1^ restoring miR-139-5p levels with a miRNA mimic, for example in combination with G-CSF, could enhance the effect of polychemotherapy especially in the treatment of CN-AML patients, which is currently based on cytarabine and anthracyclines.

The potent tumor suppressor effect of miR-139 in hematopoietic malignancies has largely gone unnoticed, given that there are only two reports so far linking miR-139 to AML.^5, 14^ We show for the first time that miR-139-5p is specifically downregulated in CN-AML with mutated FLT3 and acts as a strong tumor suppressor in a primary AML transplantation model. Our findings further rationalize the development of a miR-139 mimic as a treatment option for AML.

## Figures and Tables

**Figure 1:**
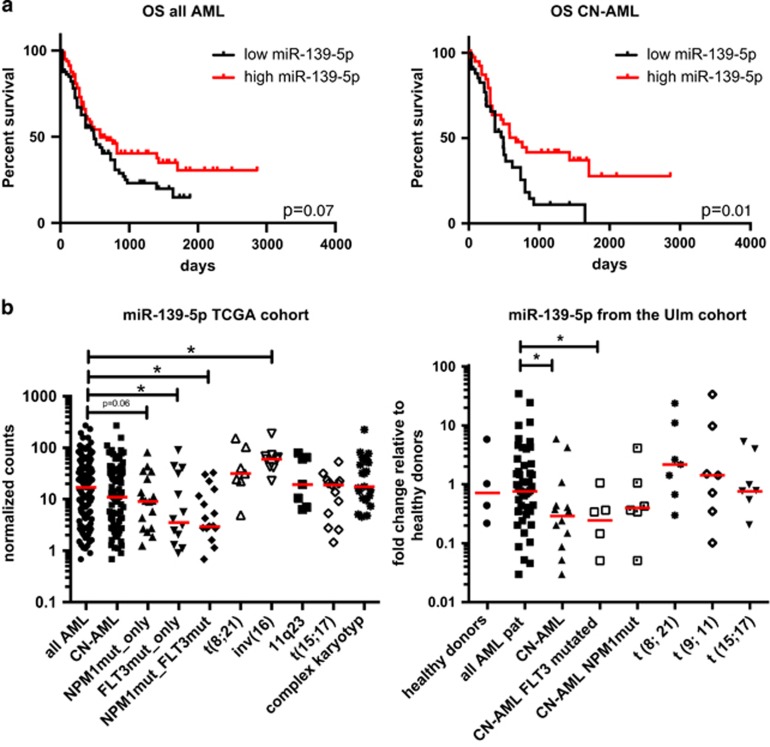
Genetic and clinical associations of miR-139-5p in adult AML. **(a)** Left panel overall survival of all analyzed AML patients dichotomized to the median expression levels of miR-139-5p. Right panel overall survival of CN-AML patients dichotomized to the median expression levels of miR-139-5p (*P*=0.01, log-rank test). Figures were generated using the miRNA sequencing data of TCGA Research Network: http://cancergenome.nih.gov/. (**b)** Left panel Expression levels of miR-139-5p (all AML: *n*=139, CN-AML: *n*=93, CN-AML NPM1mut: *n*=34, CN-AML-mutated FLT3: *n*=31, 11q23 *n*=7, t(8;21): *n*=6, t(15;17): *n*=12, complex karyotype: *n*=19, inv(16): *n*=9) analyzed from the TCGA Research Network: http://cancergenome.nih.gov/. MiRNA expression levels are in reads per million (RPM). Right panel Expression levels of miR-139-5p (all AML: *n*=49, CN-AML: *n*=16, CN-AML NPM1mut: *n*=8, CN-AML-mutated FLT3: *n*=6, t(8;21): *n*=7, t(9;11): *n*=7, t(15;17): *n*=7, healthy donors: *n*=4) in AML patients (Ulm cohort) measured by qRT-PCR. qRT-PCR was performed with Taqman miRNA Assay (Applied Biosystems, Germany) following the manufacturer's protocol. miRNA assay results were normalized to the abundance of RNU6B. The expression is presented as fold change relative to the expression of miR-139-5p in bm cells of healthy donors. The median is shown as horizontal red line for each group. Pairwise comparisons were performed using Mann–Whitney *U*-test.

**Figure 2:**
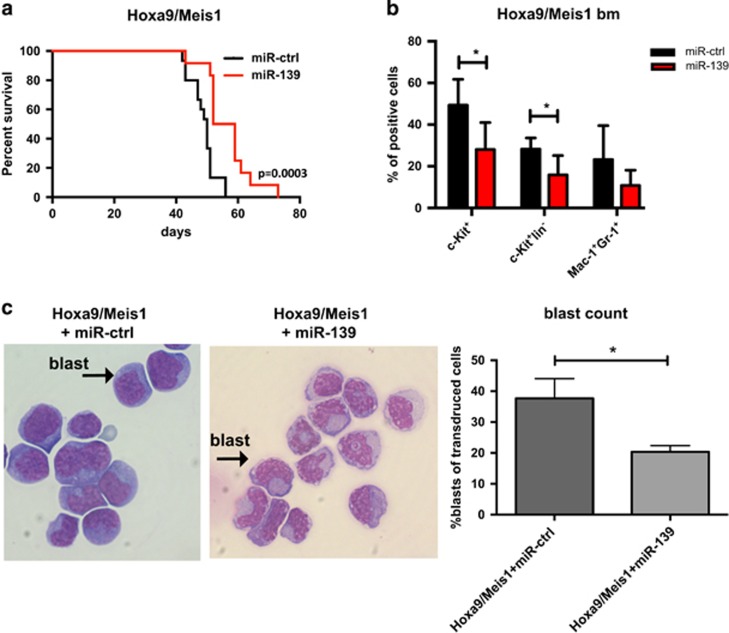
Restoration of miR-139-5p expression delays Hoxa9/Meis1-mediated leukemogenesis. (**a)** Kaplan–Meier analysis of survival of mice transplanted either with Hoxa9/Meis1/ctrl (*n*=15) or Hoxa9/Meis1/miR-139-5p (*n*=12). The graph represents a summary of two independent experiments with two biological Hoxa9/Meis1/replicates. All transplanted mice succumbed to leukemia. Mice transplanted with Hoxa9/Meis1/miR-139 developed leukemia significantly (*P*=0.0003 log-rank test) slower. (**b**)Immunophenotypic comparison by flow cytometric analysis of bm cells from deceased mice transplanted with Hoxa9/Meis1/miR-139-5p compared with their respective Hoxa9/Meis1/ctrl transplanted cohort. Donor-derived (CD45.1-positive), GFP-positive cells were stained for c-Kit, CD11b, Gr-1 as well as for a cocktail of lymphoid markers (CD4, CD8a, CD19 and CD45R). (**c**) Left panel. Changes in morphology analyzed by light-field microscopy of Wright-Giemsa-stained bm cells of deceased leukemic Hoxa9/Meis1/ctrl and Hoxa9/Meis1/miR-139 mice. Bm cells overexpressing Hoxa9/Meis1/miR-139 show a more differentiated morphology based on their nucleus shape and structure. A 100 × magnification of a representative field is shown. Right panel. Blast counts on bm from mice succumbed from Hoxa9/Meis1/miR-ctrl or Hoxa9/Meis1/miR-139 (engraftment of transduced cells between 94 and 99%). Pairwise comparisons were performed using Student's *t*-test.
