# Supplementary figures and images for: MiR-139-5p is a potent tumor suppressor in adult acute myeloid leukemia
Source: Blood Cancer J. 2016 Dec 9;6(12):e508–. doi: 10.1038/bcj.2016.110 (PMC5223146; doi:10.1038/bcj.2016.110)

Supplementary Figure S1

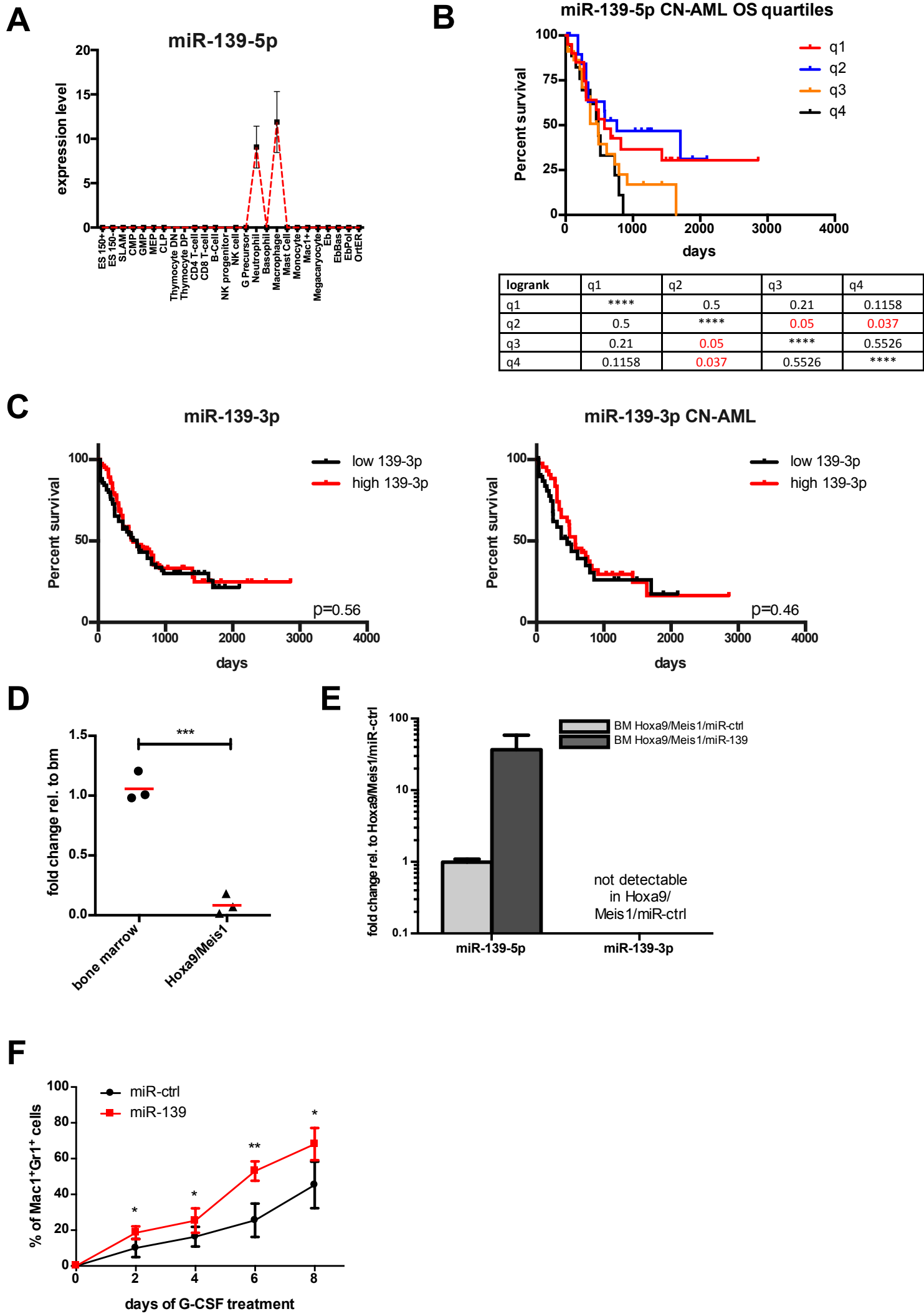

Supplement: Supplementary Figure 1 [file bcj2016110x1.pdf]
