# Supplementary material for: MiR-139-5p is a potent tumor suppressor in adult acute myeloid leukemia
Source: Blood Cancer J. 2016 Dec 9;6(12):e508–. doi: 10.1038/bcj.2016.110 (PMC5223146; doi:10.1038/bcj.2016.110)
Supplement: Supplementary Figure Legend [file bcj2016110x2.docx]

**Supplementary Figure 1: A** Microfluidic qRT-PCR expression analysis of miR-139 in 26 distinct subpopulations representing the murine hematopoietic hierarchy adapted from Petriv, Kuchenbauer et al.^14^ **B** Overall survival of CN-AML patients splitted according to miR-139-5p expression levels into quartiles and compared each quartile to the others (q1, highest expressors; q4, lowest expressors). **C** **left panel** Overall survival of all analyzed AML patients dichotomized to the median expression levels of miR-139-3p.**right panel** Overall survival of CN-AML patients dichotomized to the median expression levels of miR-139-3p**.** Figures were generated using miRNA sequencing data of TCGA Research Network: <http://cancergenome.nih.gov/>. **D** Expression analysis of miR-139-5p in Hoxa9/Meis1 cells compared to non-transformed wild-type bm cells by qRT-PCR. MiR-139-5p expression is presented as fold change relative to miR-139-5p expression in untransduced bm cells. **E** Expression analysis of miR-139-5p and miR-139-3p in bm cells of deceased Hoxa9/Meis1 mice by qRT-PCR. Expression levels in Hoxa9/Meis/miR-139 bm cells are presented as fold change relative to expression levels in Hoxa9/Meis1/miR-ctrl bm cells. No expression of miR-139-3p was detectable in Hoxa9/Meis1/miR-ctrl bm cells (n=3). QRT-PCR for D+E was performed following the manufacturer’s protocol for TaqMan Advanced miRNA Assay. (Applied Biosystems, Germany). MiR-186-5p and miR-423-5p were validated as the most stably expressed control genes. MiRNA assay results were normalized to the geometric mean of abundance of miR-186-5p and miR-423-5p ^15^. **F** Differentiation kinetics of G-CSF treated 32D cells ectopically expressing miR-139-5p (n=4) or empty control vector (miR-ctrl, n=7). Percentages of CD11b and Gr-1 double positive cells indicate granulocytic differentiation. Error bars represent mean ± SD. Pairwise comparisons were performed using Student´s t-test.
